# Effects of Walking Aids on Knee Adduction Moment After Total Knee Arthroplasty: A Gait Analysis Study

**DOI:** 10.7759/cureus.80828

**Published:** 2025-03-19

**Authors:** Shomaru Ito, Tatsuya Igawa, Shinno Iijima, Akihiro Ito, Ryunosuke Urata, Riyaka Ito, Hiroto Takahashi, Kosuke Suzuki, Mika Toda, Tsuyoshi Hara, Hitoshi Maruyama

**Affiliations:** 1 Department of Physical Therapy, School of Health Sciences, International University of Health and Welfare, Otawara, JPN; 2 Department of Physical Therapy, School of Health Sciences, International University of Health and Welfare, otawara, JPN; 3 Department of Physical Therapy, Graduate School, International University of Health and Welfare, Otawara, JPN; 4 Innovative Rehabilitation Center, New Spine Clinic, Tokyo, JPN; 5 Department of Rehabilitation, Yamagata Saisei Hospital, Yamagata, JPN; 6 Department of Physical Therapy, Faculty of Medicine, Fukuoka International University of Health and Welfare, Fukuoka, JPN

**Keywords:** gait analysis, knee adduction moment, knee osteoarthritis, total knee arthroplasty, walking aids

## Abstract

Background: Total knee arthroplasty (TKA) is a common intervention for treating end-stage knee osteoarthritis (OA). Postoperatively, altered biomechanics may increase the risk of OA progression in the contralateral knee. Knee adduction moment (KAM) is a key indicator of medial knee joint loading and is associated with OA progression. Walking aids are often prescribed to reduce joint stress during walking; however, their comparative effects on KAM in patients undergoing TKA remain unclear. This study aimed to evaluate the effects of different walking aids (double canes [D-canes], rollators without forearm support [rollators], and rollators with forearm support [F-rollators]) on KAM peak and impulse in both the operated and contralateral knees of patients after TKA using gait analysis.

Methodology: A cross-sectional study was conducted on eight women who underwent unilateral TKA. Participants walked under four conditions: no aid, D-canes, rollators, and F-rollators. A three-dimensional motion capture system measured the lower limb kinematics and kinetics. The primary outcomes were KAM peak and impulse for both knees. Secondary outcomes included ground reaction force in the vertical direction (GRF Z), forward trunk tilt angle, and lower limb joint angles. A two-way repeated-measures measures analysis of variance (ANOVA) was used for statistical comparisons.

Results: Walking aids significantly reduced the KAM peak and impulse in both the operated and contralateral knees compared with unaided walking (*P* < 0.001). In the operated knee, the KAM peak decreased by approximately 20% with all aids, whereas in the contralateral knee, reductions were 6% for D-canes, 16% for rollators, and 24% for F-rollators. Rollators and F-rollators more effectively reduced contralateral knee loading than D-canes (*P* < 0.001). A significant negative correlation was found between trunk forward tilt angle and GRF Z (ρ= -0.74, *P* < 0.001), suggesting that increased forward lean with rollators and F-rollators enhanced lower limb offloading. No significant changes were observed in knee varus angle or walking speed across conditions.

Conclusions: Walking aids, particularly F-rollators, effectively reduce KAM and offload the contralateral knee in patients after TKA. These findings suggest potential benefits for patients at risk of OA progression in non-operated knees.

## Introduction

Osteoarthritis (OA) is a disease characterized by age-related degeneration and subsequent pathological changes in the surrounding joint tissues. More than 300 million people worldwide are estimated to have OA [1]. Knee OA, in particular, significantly impairs walking ability and activities of daily living (ADL) in older adults due to pain, range of motion (ROM) limitation, and muscle weakness [2,3]. If these declines in physical function are not addressed, they may lead to increased healthcare costs, a higher likelihood of requiring long-term care, and reduced healthy life expectancy [4]. Therefore, early intervention in knee OA is essential.

Total knee arthroplasty (TKA) is a common surgical treatment for knee OA, involving the replacement of degenerated joint surfaces with artificial components and bone realignment. This procedure effectively reduces pain and improves ADL and quality of life (QoL) [5,6]. However, long-term studies have shown that approximately 60% of patients who undergo unilateral TKA develop contralateral knee OA, with varying severity depending on the study population and follow-up period [7]. Additionally, approximately 30% of these patients may require contralateral TKA within five years [8].

Mechanical stress exerted on the knee joint is a primary factor in the progression of knee OA [9]. Knee adduction moment (KAM) during walking is frequently used as an indicator of mechanical stress on the knee joint [10]. Miyazaki et al. demonstrated that even a slight increase in the maximum KAM (KAM peak) was significantly associated with the progression of knee OA severity based on radiographic findings (Kellgren-Lawrence grading system) [11]. These findings suggest that an increased knee medial compartment load resulting from elevated KAM may contribute to the progression of knee OA. Therefore, the assessment of KAM is clinically significant. Subsequent studies suggest that an increase in KAM may result in an increased load on the medial compartment of the knee [12]. However, gait analysis after TKA shows a decrease in the KAM peak on the surgical side, whereas the contralateral side often exhibits an increase in the KAM peak [13-15]. These findings suggest that the contralateral side of the knee joint is prone to excessive mechanical stress after TKA.

Given this, post-TKA rehabilitation should not only focus on the operated knee but also aim to prevent disease progression and provide care for the contralateral knee. One recommended strategy is the use of walking aids, such as canes and rollators [16,17]. Extant research has shown that using walking aids can reduce the KAM peak by approximately 10%-20% in both older adults with OA and patients with knee OA [18-20]. However, many of these studies focused on biomechanics and KAM during a single-leg stance. Few studies have quantitatively compared the changes in KAM in both knees of patients after TKA. Furthermore, no studies have assessed the effects of using double canes (D-canes; use of two canes) or rollators with or without forearm support on KAM reduction.

Based on these findings, the purpose of this study was to analyze the gait of patients after TKA using three types of walking aids: D-canes, rollators without forearm support (rollators), and rollators with forearm support (F-rollators). The primary objective was to compare and evaluate the effects of these walking aids on mechanical stress, particularly in terms of KAM, in both the operative and contralateral knees. This study specifically examined the impact of different walking aids on the KAM peak and impulse, assessing the effectiveness of each aid. The choice of walking aid may influence the degree of KAM reduction in patients after TKA, with F-rollators being the most effective option. The results of this study are expected to provide clinical guidance for selecting appropriate walking aids for TKA rehabilitation, preventing knee OA progression, and improving ADL and QoL.

## Materials and methods

Participants

Eight women (16 knees) who underwent unilateral TKA at a single institution in Japan between April 2013 and April 2019 were included in this study. The inclusion criteria were being at least three months postoperative and independently performing daily activities, either walking unassisted or using a cane. The exclusion criteria included prior orthopedic surgery other than TKA or cardiovascular or central nervous system diseases affecting walking.

Data collection

Demographic data, including age, height, weight, body mass index (BMI), TKA design, surgical approach, postoperative complications, intraoperative knee ROM, and Kellgren-Lawrence (K-L) grading system for both the operated and contralateral knees, were obtained from electronic medical records and questionnaires. The K-L grading was assessed by orthopedic surgeons based on weight-bearing knee radiographs.

Physical function data, including knee passive ROM (flexion and extension), knee extension strength, and knee pain, were collected and assessed using a visual analog scale (VAS). The ROM was measured in 5° increments using a plastic goniometer (Suzuki Medical Inc., Machida, Japan) [21]. Knee extension strength was measured using a handheld dynamometer (Mobi MT-100; Sakai Medical Co., Ltd., Shinjuku, Japan) following Wikholm’s method [22], with the highest value recorded in two trials. Pain was assessed using a 0-100 mm VAS, where 0 indicated *no pain* and 100 represented *maximum pain*. Participants rated the pain experienced while walking over the past week [23].

The primary outcome was the KAM peak. Secondary outcomes included joint angles and moments of other lower limb joints, forward trunk tilt angles, and spatiotemporal gait characteristics, such as walking speed and stride length. The stance phase was divided into the early and late phases, and the KAM peak was determined for each phase. The KAM impulse, which represents the integral of the KAM over the stance phase, was calculated using the method described by Chang et al. [24]. The stance phase, defined from heel-strike to toe-off, was identified based on ground reaction force (GRF) data.

**Figure 1:**
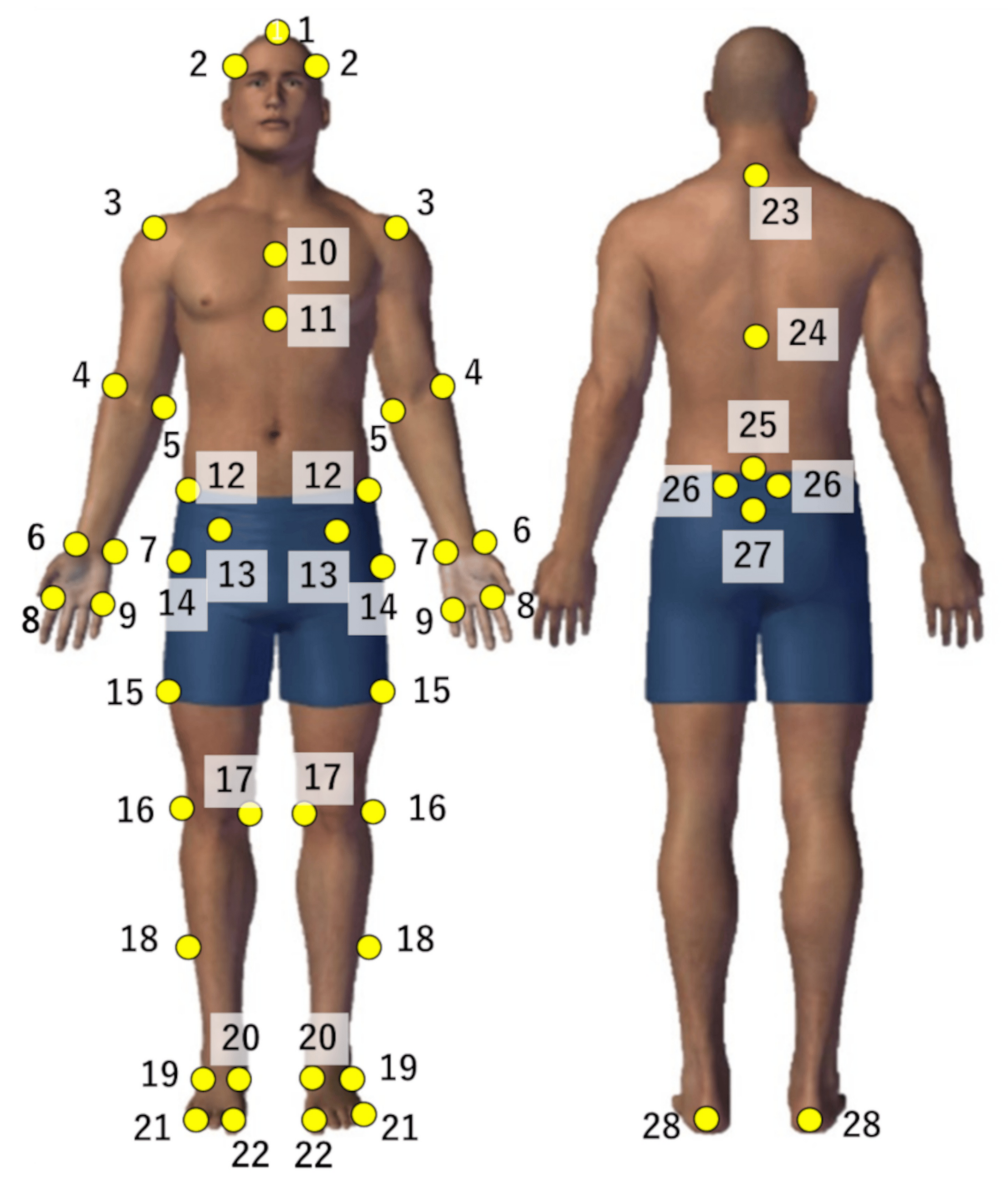
Forty-nine reflective markers. 1・2: Top of head and above auricle, 3・4・5: Acromion, humerus lateral and medial epicondyles, 6・7: Radial and ulnar styloid process, 8・9: 2nd and 4th metacarpal bones, 10・11: Manubrium and xiphoid process, 12・13・14: Iliac crest, anterior superior iliac spine and superior aspect of the greater trochanter, 15: Middle of the thigh, 16・17: Inside and outside of the knee joint, 18: Middle of the shank, 19・20: Lateral and medial malleolus, 21・22: first and fifth metatarsal heads, 23・24・25: seventh cervical vertebra, 10th thoracic vertebra and fifth lumbar vertebra, 26: Posterior superior iliac spine, 27: Sacrum, 28: Calcaneus Image credits: Shomaru Ito.

A three-dimensional motion analysis system comprising 10 infrared cameras (VICON MX, Vicon Motion Systems, Oxford, UK) and six force plates (AMTI, Watertown, MA; Kistler, Winterthur, Switzerland) was used to obtain the kinematic and kinetic data. The cameras and force plates were set to sampling frequencies of 100 Hz and 1000 Hz, respectively. Reflective markers (14 mm diameter) were placed at 49 anatomical locations based on previous studies (Figure 1) [25].

**Figure 2:**
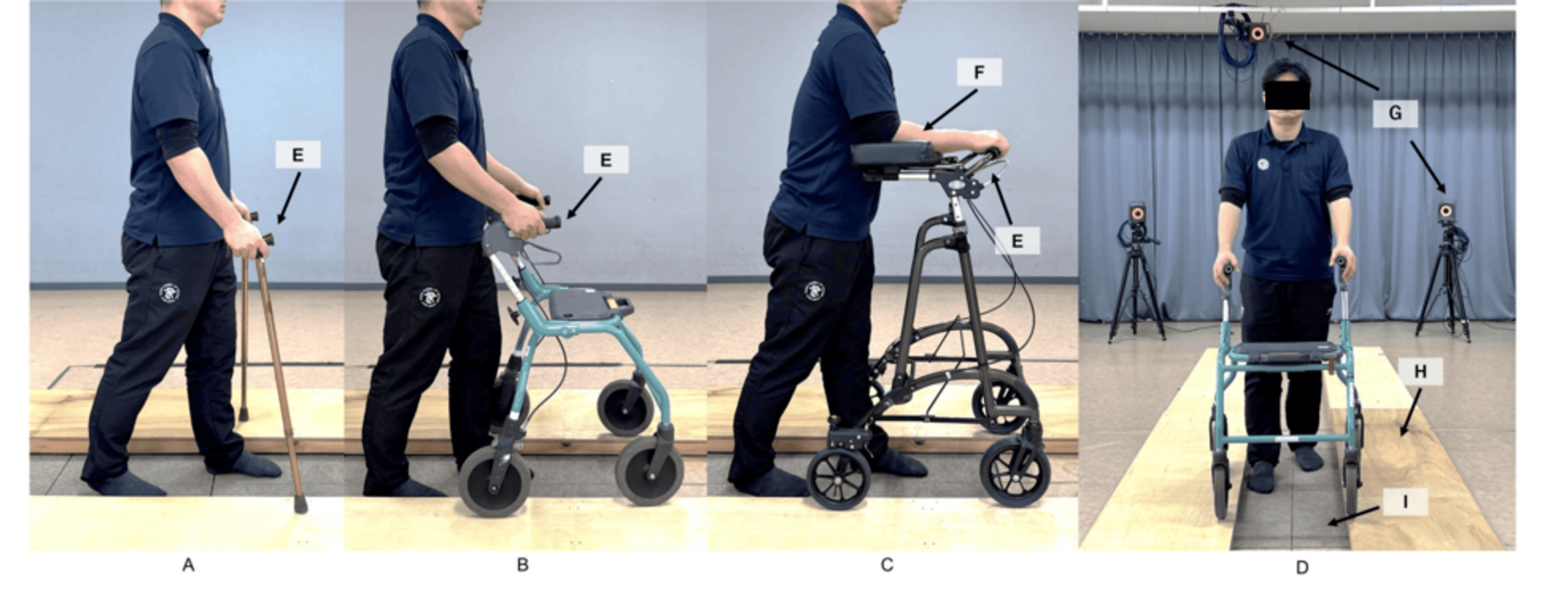
Walking aids, walkingways, and measurement environments. (A) Double canes (Welfan Inc., Yume Life Stick), (B) Rollators without forearm support (Invacare Ltd., Dolomite Opal 2000), (C) Rollators with forearm support (Wel-Partners Co., Ltd., Labbito WA-5), (D) Rollators with forearm support (Wel-Partners Co., Ltd., Rabbit WA-5), (E) Walking paths and measurement environments (10m walking path, 10 infrared cameras, and 6 force plates), (F) Hand grip, (G) Forearm support, (H) Infrared cameras, (I) Boards (for walking aids), (J) Force plates.

The measurement environment was a 10 m straight walking way. Walking aids were positioned on the boards to measure only the GRF of the lower limbs (Figure 2). A metronome was used to control the walking speed at a walking rate of 100 steps per minute [26]. The walking conditions included no aids (walking without walking aids), double canes (D-canes; Yume Life Stick Basic Type, Welfare Inc., Osaka, Japan), and rollators without forearm support (Rollators, Dolomite Opal 2000, Invacare Holdings Co., OH), and rollators with forearm support (F-rollators, Wel-Partners Co., Rabbit WA-5) (Figure 2). Each condition was repeated three times, and the mean values from the three trials were used for the analysis. The handle heights of the canes and rollators were adjusted to the radial styloid process while standing [20,27], whereas the forearm-supporting rollator handle was adjusted to the elbow height [28]. The participants received instructions on how to use each walking aid and completed five practice trials before data collection.

Data analysis

Coordinate data from the infrared reflective markers and GRF data from the force plates were processed using Visual 3D version 6 (C-Motion, Germantown, MD). A second-order recursive Butterworth filter was applied, with cutoff frequencies set at 6 Hz for the coordinate data and 18 Hz for the GRF data following Winter’s methodology [29]. The joint angles were calculated using the Euler angles, and the joint moments were determined through an inverse dynamics analysis using a link-segment model. The segment mass ratios, center of mass, and moments of inertia required for the joint moment calculations were obtained from previous studies [30]. The global coordinate system was defined as follows: X-axis (left-right), Y-axis (anterior-posterior), and Z-axis (vertical) with positive directions to the left, front, and upward, respectively.

Statistical analysis

VAS scores, ROM, and knee extension strength were analyzed using the Mann-Whitney U test and Wilcoxon signed-rank test, respectively. Kinematic and kinetic parameters were analyzed using two-way repeated-measures analysis of variance (ANOVA) with walking aids and lower limbs as independent variables. When significant main effects were detected, multiple comparisons were performed using the Bonferroni method. For significant interactions, simple main-effects tests were conducted. The relationship between the forward trunk tilt angle and the vertical component of the GRF (GRF Z) was analyzed using Spearman’s rank correlation coefficient. Statistical analyses were conducted using SPSS version 29 (IBM Corp., Armonk, NY), with significance set at *P* < 0.05.

Ethical approval

This study was approved by the Ethics Committee of the International University of Health and Welfare (17-Io-143). Informed consent was obtained from all the participants by the Declaration of Helsinki.

## Results

Table 1 shows that the knee flexion angle on the operative side was significantly reduced compared with the contralateral side (operative side: 118.1° ± 12.8°; contralateral side: 134.4° ± 9.8°; *P* < 0.05).

**Table 1:** Physical function data and demographic data (n = 8). Mean ± standard deviation. *P < 0.05. K-L grade, Kellgren-Lawrence grading system; POD, postoperative day; CR, cruciate retaining; PS, posterior stabilized; BCR, bicruciate retaining; Medial, medial parapatellar approach; subvastus, subvastus approach; VAS, visual analog scale (knee pain during gait)

	Operation	Contralateral
Age (years)	72.8±7.2	-
Height (cm)	150.4±6.6	-
BMI (kg/m²)	60.9±13.1	-
K-L grade (grades: n)	4:8	0:2
		2:3
		3:2
		4:4
POD (months)	34.9 ± 16.8	-
Designs (designs: n)	CR: 1	-
	PS: 5	-
	BCR: 2	-
Approach (approach: n)	Medial: 3	-
	Subvastus: 5	-
Intraoperative knee flexion ROM (degrees)	127.9 ± 9.1	-
Intraoperative knee extension ROM (degrees)	0 ± 0	-
VAS (mm)	58.8 ± 117.7	77.5 ± 99.2
Knee flexion angle (degrees)	118.1 ± 12.8*	134.4 ± 9.8*
Knee extension angle (degrees)	-4.4 ± 5.0	-5.6 ± 9.4
Knee extension strength (kgf/kg)	0.43 ± 0.18	0.41 ± 0.17

Spatiotemporal gait characteristic data did not reveal any significant differences (Table 2).

**Table 2:** Spatiotemporal gait characteristics data (n = 8). Mean ± standard deviation. D-canes: Use of two canes Rollators: Rollators without forearm support F-rollators: Rollators with forearm support

	No aids	D-canes	Rollators	F-rollators
Velocity (m/s)	0.97 ± 0.12	0.87 ± 0.13	0.94 ± 0.13	0.92 ± 0.13
Stride length (m)	1.07 ± 0.09	1.10 ± 0.10	1.10 ± 0.09	1.10 ± 0.11

The results for the joint moments, GRFs, and joint angles are listed in Table 3.

**Table 3:** Kinematic and kinetic data (n = 8). Mean ± standard deviation *, †, ‡, §, ||, ¶, ⁋ : *P* < 0.05. **, ††, ‡‡, §§, ||||, ¶¶, ⁋⁋ : *P* < 0.001. *No aids vs. D-canes. †No aids vs. rollators. ‡No aids vs. F-rollators. §D-canes vs. rollators ||D-canes vs. F-rollators. ¶Rollators vs. F-rollators. ⁋Operation vs. contralateral. **No aids vs. D-canes. ††No aids vs. rollators. ‡‡No aids vs. F-rollators. §§D-canes vs. rollators. ||||D-canes vs. F-rollators. ¶¶Rollators vs. F-rollators. ⁋⁋Operation vs. contralateral. D-canes: use of two canes; Rollators: rollators without forearm support; F-rollators: rollators with forearm support KAM peak and hip abduction moment in the early stance phase showed interaction; therefore, a simple main effect test was performed. KAM, knee adduction moment; GRF Z, the vertical component of ground reaction force; GRZ Y, the medial-lateral component of ground reaction force

			No aids	D-canes	Rollators	F-rollators	Main effect (walking aids) *P*-value	Main effect (lower limbs) *P*-value	Interaction (walking aids × lower limbs) *P*-value
KAM peak (Nm/kg) ; adduction +	Early stance phase^**,††,‡‡^^,^^⁋^	Operation	0.41 ± 0.07^⁋^	0.32 ± 0.06**^,^^⁋^	0.31 ± 0.07^††,^^⁋^	0.33 ± 0.07^‡‡^	<0.001	0.015	0.001
		Contralateral	0.52 ± 0.12	0.49 ± 0.10	0.43 ± 0.09^††,§§^	0.39 ± 0.11^‡‡,||^^||^			
	Late-stance phase^**,†,‡‡^	Operation	0.29 ± 0.10	0.18 ± 0.09	0.21 ± 0.08	0.18 ± 0.09	<0.001	0.06	0.36
		Contralateral	0.36±0.11	0.30±0.07	0.28±0.08	0.26±0.09			
KAM impulse (s*Nm/kg) ; abduction +	Stance phase^**,††,‡‡,^^⁋^	Operation	0.24 ± 0.07	0.18 ± 0.06	0.18 ± 0.05	0.18 ± 0.06	<0.001	0.012	0.23
		Contralateral	0.31 ± 0.07	0.28 ± 0.05	0.26 ± 0.05	0.25 ± 0.06			
Hip abduction moment (Nm/kg); abduction +	Early stance phase^*,††,‡‡^	Operation	0.79 ± 0.10	0.65 ± 0.13*	0.65 ± 0.12^††^	0.64 ± 0.12^‡^	<0.001	0.047	0.64
		Contralateral	0.80 ± 0.14	0.74 ± 0.12	0.67 ± 0.10^††^	0.61 ± 0.12^‡‡,||^			
	Late-stance phase^*,†,‡^	operation	0.60 ± 0.17	0.44 ± 0.15	0.46 ± 0.15	0.44 ± 0.17	<0.001	0.92	0.624
		contralateral	0.55±0.09	0.47±0.13	0.49±0.11	0.45±0.12			
Knee varus angle (degrees); varus +	Early stance phase^⁋⁋^	Operation	0.0 ± 3.0	0.1 ± 2.6	-0.1 ± 3.0	0.1 ± 2.4	0.76	0.005	0.007
		Contralateral	4.5 ± 2.7	4.5 ± 2.7	4.4 ± 2.4	4.5 ± 2.7			
	Late-stance phase^⁋^	Operation	-0.2 ± 2.3	-0.5 ± 1.9	-0.4 ± 2.4	-0.1 ± 1.7	0.125	0.006	0.86
		Contralateral	3.6 ± 2.8	3.6 ± 2.7	3.6 ± 2.8	3.9 ± 3.1			
Hip adduction angle (degrees); adduction +	Early stance phase^⁋^	Operation	4.4 ± 2.0	4.1 ± 2.0	4.0 ± 1.7	4.1 ± 1.8	0.76	0.045	0.25
		Contralateral	1.9 ± 1.6	2.5 ± 1.9	2.3 ± 1.8	2.6 ± 1.6			
	Late-stance phase	Operation	4.1 ± 1.8	3.6 ± 1.7	3.9 ± 1.3	3.5 ± 1.4	0.82	0.20	0.068
		Contralateral	1.8 ± 1.4	2.0 ± 2.0	2.4 ± 2.3	2.4 ± 2.0			
Trunk forward tilt angle (degrees); forward +	Early stance phase^††,§§,‡‡,|| ||,¶^	Operation	0.6 ± 3.9	4.3 ± 4.2	8.5 ± 4.5	21.0 ± 8.9	<0.001	0.86	1.00
		Contralateral	0.4 ± 4.0	3.6 ± 5.0	8.3 ± 4.9	20.7 ± 9.1			
	Late-stance phase^††,§§,‡‡,|| ||,¶¶^	Operation	0.4 ± 3.8	2.8 ± 3.6	8.3 ± 4.7	14.7 ± 8.4	<0.001	1.00	0.99
		Contralateral	0.4 ± 3.8	2.6 ± 2.6	8.1 ± 4.7	14.3 ± 8.3			
GRF Z (N/(kg・9.8)); upward +	Early stance phase^††,§§,‡‡,|| ||,¶¶^	Operation	0.95 ± 0.05	0.93 ± 0.07	0.83 ± 0.05	0.78 ± 0.08	<0.001	1.00	0.86
		Contralateral	1.00 ± 0.08	0.95 ± 0.06	0.88 ± 0.05	0.81 ± 0.07			
	Late stance phase^††,‡‡,||,¶^	Operation	0.96 ± 0.05	0.92 ± 0.08	0.89 ± 0.05	0.79 ± 0.08	<0.001	0.99	0.70
		Contralateral	0.98 ± 0.06	0.93 ± 0.07	0.89 ± 0.06	0.81 ± 0.11			
GRF Y (N/(kg・9.8)); left +	Early stance phase	Operation	0.08 ± 0.03	0.08 ± 0.03	0.06 ± 0.02	0.05 ± 0.03	0.23	0.23	0.33
		contralateral	0.08±0.03	0.08±0.02	0.08±0.04	0.08±0.04			
	Late-stance phase	Operation	-0.09 ± 0.05	-0.10 ± 0.03	-0.11 ± 0.04	-0.10 ± 0.04	0.32	0.25	0.54
		Contralateral	-0.12 ± 0.03	-0.12 ± 0.03	-0.11 ± 0.04	-0.08 ± 0.02			

The KAM peak in the early stance phase exhibited significant main effects and interactions related to walking aids and lower limb factors. Post hoc multiple comparisons revealed that the KAM peak was significantly lower in all walking-aid conditions than in the no-aid condition (*P* < 0.001). In addition, simple main effect tests were conducted based on the observed interactions. These tests indicated that the KAM peak on the operative side was significantly reduced with all walking aids compared with no aids (no aids: 0.41 ± 0.07 Nm/kg, D-canes: 0.32 ± 0.06 Nm/kg, rollators: 0.31 ± 0.07 Nm/kg, F-rollators: 0.33 ± 0.07 Nm/kg, *P* < 0.001). On the contralateral side, the KAM peak was significantly lower with rollators and F-rollators compared with no aids and D-canes (no aids: 0.52 ± 0.12 Nm/kg, D-canes: 0.49 ± 0.10 Nm/kg, rollators: 0.43 ± 0.09 Nm/kg, F-rollators: 0.39 ± 0.11 Nm/kg, *P* < 0.001). Furthermore, the peak KAM on the operative side was significantly lower than that on the contralateral side with no aid, D-canes, or rollators (*P* < 0.05). In the late stance phase, the KAM peak showed a significant main effect of the walking aids, with all walking aids significantly reducing the KAM peak compared with no aids (*P* < 0.05).

The KAM impulse demonstrated significant main effects for walking aids and lower limb factors. Post hoc multiple comparisons revealed that the KAM impulse was significantly lower in all walking-aid conditions than in the no-aid condition (*P* < 0.001). The KAM impulse on the operative side (no aids: 0.24 ± 0.07 s*Nm/kg, D-canes: 0.18 ± 0.06 s*Nm/kg, rollators: 0.18 ± 0.05 s*Nm/kg, F-rollators: 0.18 ± 0.05 s*Nm/kg) was significantly lower than on the contralateral side (no aids: 0.31 ± 0.07 s*Nm/kg, D-canes: 0.28 ± 0.05 s*Nm/kg, rollators: 0.26 ± 0.05 s*Nm/kg, F-rollators: 0.25 ± 0.06 s*Nm/kg) (*P* < 0.05).

The hip abduction moment in both stance phases exhibited significant effects on the walking aid factor. Additionally, a significant interaction was observed during the early stance phase. Post hoc multiple comparisons indicated that the hip abduction moment with all walking aids in both stance phases was significantly lower than that without aids (*P* < 0.05). Simple main effect tests showed that on the operative side, the hip abduction moment with all walking aids was significantly reduced compared to that with no aid (*P* < 0.05). On the contralateral side, the hip abduction moments with rollators and F-rollators were significantly lower than those without aids, and the moment with F-rollators was significantly lower than that with D-canes (*P* < 0.05). In the late-stance phase, all walking aids were associated with a significant reduction in the hip abduction moment compared with no aid (*P* < 0.05).

The knee valgus angle showed a significant main effect of the lower limb factors in both stance phases. Multiple comparisons indicated that the knee valgus angle on the contralateral side was significantly larger than that on the operated side (*P* < 0.05).

The hip abduction angle demonstrated a significant main effect of the lower limb factors in the early stance phase. Multiple comparisons indicated that the knee valgus angle on the contralateral side was significantly larger than that on the operated side (*P* < 0.05).

The forward trunk tilt angle exhibited a significant main effect of the walking aid factors in both the early and late stance phases. Multiple comparisons indicated that the forward trunk tilt angle with rollators was significantly greater than that with no aids or D-canes, and F-rollators showed a significant increase compared with all other conditions (*P* < 0.05). Table 4 shows the negative correlation between the forward trunk tilt angle and GRF Z (ρ = -0.74, *P* < 0.001).

**Table 4:** Correlation between forward trunk tilt angle and GRF Z (n = 8). GRF Z, the vertical component of ground reaction force

	GRF Z		
Forward trunk tilt angle	ρ = -0.77, *P* < 0.001		

The GRF Z showed a significant main effect on walking aids in both the early and late stance phases. Multiple comparisons revealed that the GRF Z in the early stance phase with rollators was significantly lower than that with no aids and D-canes and that the GRF Z with F-rollators was significantly lower than that with all other conditions (*P* < 0.001). In the late-stance phase, the GRF Z with rollators was significantly lower than that without aids, and the GRF Z with F-rollators was significantly lower than that under all other conditions (*P* < 0.05).

## Discussion

This study aimed to clarify the changes in KAM in patients undergoing TKA using different walking aids by analyzing their gait.

Changes in KAM with walking aids

This study found significant main effects of the type of walking aid on both the KAM peak and KAM impulse. The KAM peak represents the instantaneous load on the knee joint, whereas the KAM impulse reflects the average load on the knee joint during the stance phase [24]. Therefore, assessing both parameters is essential when evaluating mechanical stress on the knee joint. While many previous studies have focused on KAM peaks [18,19,31], reports on the KAM impulse are limited, with Simic et al. being the only study to investigate the KAM impulse for the use of a single cane [20]. This study is the first to evaluate both KAM peak and KAM impulse using multiple walking aids, providing new insights.

All walking aids (D-canes, rollators, and F-rollators) contributed to the reduction of both the KAM peak and KAM impulse, suggesting a potential reduction in mechanical stress on the knee joint in patients with TKA. The KAM peak on the operative side decreased by approximately 20% (D-canes, 22%; rollator, 23%; F-rollator, 18%) and on the contralateral side by 15% (D-canes, 6%; rollator, 16%; F-rollator, 24%). These findings are consistent with previous reports in healthy older adults and patients with knee OA, where the peak KAM was reduced by 10%-20% [18-20], suggesting the possible effectiveness of walking aids for patients with TKA.

Changes in KAM with different types of walking aids

On the operative side, both early and late stance phase KAM peaks were significantly reduced with all walking aids compared with no aids. On the contralateral side, the rollators and F-rollators showed a more significant reduction in the KAM peak during the early stance phase than the D-canes and no aids. Similar results were obtained for the KAM impulses. Therefore, all walking aids used in this study may have reduced KAM after TKA. However, for the contralateral KAM peak, D-canes did not result in a reduction, whereas rollators and F-rollators significantly reduced the KAM more than D-canes. Therefore, rollators and F-rollators may be more effective in reducing contralateral KAM. In addition, all conditions except for the F-rollators showed significant differences in the KAM peaks for both sides. These results suggest that F-rollators may be the most useful walking aids for reducing both-sided KAM in patients undergoing TKA.

Factors in reduced KAM with walking aids

The joint moments were calculated using the GRF and lever arms [30]. In the KAM, the varus angle of the knee influences the lever arm [32,33]. This study observed no significant effect of walking aids on the knee varus angle and the GRZ Y, but a significant effect on the GRF Z. The results suggest that walking aids may alter KAM by reducing GRF Z. Post hoc comparisons showed that both rollators and F-rollators significantly reduced GRF Z compared with the D-cane. This reduction in GRF Z may be a key factor contributing to the greater reduction in the KAM peak observed with the rollators and F-rollators than with the D-canes. Therefore, the reduction in the GRF Z appears to play a critical role in explaining why the rollators and F-rollators were more effective at reducing the KAM peak than the D-canes. Weight-bearing on walking aids helps reduce the lower limb load, and the GRF Z is influenced by this load [27]. It is possible that the rollators and F-rollators, with their four-wheel support, may have reduced the load more effectively than the D-canes, which only provided two points of support.

Relation between changes in posture and floor reaction force with walking aids

Alkjær et al. reported that the use of walking aids may change posture [27]. In this study, the forward trunk tilt angle with rollators and F-rollators increased significantly compared with that with no aids and D-canes. A forward trunk tilt is necessary to bear weight on walking aids, which correlates with a reduction in GRF Z (ρ = -0.74). Therefore, walking aids may reduce the GRF Z by tilting the trunk forward, which, in turn, reduces the KAM.

Relation between walking speed and ground reaction force with walking aids

Changes in the GRF Z due to walking aids may also be influenced by walking speed [30]. Fang et al. reported a decrease in walking speed and GRF Z when patients with knee OA used walking aids [19]. Previous studies have not reached a consensus on whether the reduction in GRF Z with walking aids is primarily due to decreased walking speed or reduced lower-limb loading [19,27,34]. In this study, changes in the GRF Z were evaluated under controlled walking speeds. The results indicated that while the use of walking aids led to a reduction in GRF Z, the walking speed remained unchanged. These findings suggest that the reduction in GRF Z associated with walking aid use occurs independently of the walking speed.

Limitations and prospects of this research

This study had some limitations. First, the participants were women from a single institution, which may limit the generalizability of the findings. In addition, the sample size may have influenced the reliability of the results. Future studies should apply appropriate statistical adjustments (e.g., bootstrap methods) and conduct multicenter studies. Second, as this was a cross-sectional study, the effects of reduced KAM on patients with TKA were not directly investigated. Therefore, longitudinal studies examining ADL and QOL are required to clarify these effects. Takanokura reported that the height of rollator handgrips affects mechanical stress, indicating the need for further research to determine the optimal settings for walking aids [35]. This study showed that walking aids can reduce the KAM in patients undergoing TKA. F-rollators may be particularly useful for reducing KAM on both sides and are particularly recommended for patients with TKA with severe OA of the contralateral knee or those with decreased physical function.

## Conclusions

This study is the first to simultaneously evaluate changes in the KAM peak and KAM impulse using multiple walking aids (D-canes, rollators, F-rollators) in patients with TKA. All walking aids significantly reduced both KAM peak and KAM impulse, with F-rollators showing the greatest reduction on both sides. These results suggest that walking aids reduce knee joint mechanical stress, with F-rollators potentially being a promising option for TKA rehabilitation. The changes in KAM were likely influenced by weight-bearing on the walking aids, reducing the GRF Z, although further studies are needed to confirm this mechanism. The degree of KAM reduction varied by walking aid type, with rollators and F-rollators more effective than D-canes in reducing contralateral knee stress. These findings highlight the importance of selecting appropriate walking aids for TKA rehabilitation to prevent OA progression and improve ADL and QoL. However, caution is necessary when generalizing these results, as the sample consisted only of female patients from a single facility. Future multicenter and longitudinal studies are needed to assess long-term effects and develop personalized selection criteria based on OA severity and physical function.
